# Menaquinone synthesis genes are critical for the decolorization of acid yellow 36 in *Shewanella putrefaciens* CN32

**DOI:** 10.1128/spectrum.01996-26

**Published:** 2026-09-02

**Authors:** Jiawen Liu, Jing Yang, Aofei Sun, Hao Guan, Weibing Chen, Heng Geng, Xiaoxuan Zhang, Zhaolan Li, Di Sun, Jingrong Zhu, Zhen Dong, Cong Liu, Weijie Liu

**Affiliations:** 1 Department of Microbiology, School of Life Sciences, Jiangsu Normal University 12675, Xuzhou, Jiangsu, China; Ruhr-Universitat Bochum, Bochum, Germany

**Keywords:** menaquinone, decolorization, *Shewanella putrefaciens *CN32, CymA, *menD*

## Abstract

The anaerobic decolorization of azo dyes by *Shewanella* species relies on an intricate electron transfer network, yet the full complement of genes involved remains incompletely defined. In this study, we used mini-Tn5 transposon mutagenesis to identify genes required for the decolorization of the azo dye acid yellow 36 in *Shewanella putrefaciens* CN32. Two genes were uncovered: *menD*, encoding a key enzyme in menaquinone biosynthesis, and *lutB*, involved in lactate metabolism. Deletion of *menD* caused a nearly complete loss of decolorization capacity without affecting growth. Supplementation with vitamin K_2_ or spent supernatant from the wild-type strain restored decolorization, demonstrating that menaquinone is essential. We systematically identified the menaquinone biosynthesis gene cluster of *S. putrefaciens* CN32, including *menA*, *menB*, *menC*, *menE*, *menF*, *menG*, *menH*, and *paaI*. Deleting most of these genes severely impaired decolorization, whereas deletions of *menH*, *paaI*, or an adjacent LysR-family regulator gene had no significant effect. Furthermore, acid yellow 36 induced biofilm formation in a dose-dependent manner, and this induction was abolished in most menaquinone synthesis mutants. Acid yellow 36 did not substantially upregulate transcription of the *men* gene cluster, suggesting that the dye promotes biofilm formation through a direct mechanism rather than by enhancing menaquinone synthesis. Collectively, our results establish menaquinone as a critical component for both azo dye decolorization and biofilm formation in *S. putrefaciens* CN32, and they point to the existence of a CymA-independent electron transfer branch in this strain.

**IMPORTANCE:**

Azo dyes are environmental pollutants that can be effectively removed by *Shewanella* bacteria through anaerobic respiration. Understanding the genetic basis of azo dye decolorization is essential for improving bioremediation strategies. This study identifies menaquinone, a respiratory chain cofactor, as indispensable for the decolorization of acid yellow 36 in *Shewanella putrefaciens* CN32. By systematically mapping the menaquinone biosynthesis gene cluster, we show that an intact synthesis pathway is required for both dye reduction and biofilm formation, a lifestyle that enhances bacterial survival and metabolic activity in wastewater treatment systems. Importantly, the severe decolorization defect of menaquinone-deficient mutants contrasts with the relatively mild effect of deleting cymA, the canonical electron hub, suggesting the existence of a CymA-independent electron transfer route from menaquinone to azo dyes. These findings expand the current model of extracellular electron transfer in *Shewanella* and provide new genetic targets for engineering more efficient azo dye-degrading strains.

## INTRODUCTION

Synthetic dyes are widely used in the textile, paper, and food industries due to their rich colors, ease of preparation, and low cost (1). Among them, azo dyes, which feature an azo group as the chromophore, represent the largest and most versatile class of synthetic dyes (2, 3). Despite their irreplaceable role, wastewater generated from azo dye production causes severe environmental pollution (4, 5). Studies have shown that untreated azo dye-containing wastewater disrupts the ecological balance of water bodies and soil and inhibits the growth of aquatic organisms and crops (6, 7). Moreover, azo dyes are carcinogenic and teratogenic, and can accumulate in the human body through the food chain, ultimately endangering human health (8, 9). Current methods for removing azo dyes from wastewater include physical, chemical, and biological approaches (10, 11). Biological treatment, which employs microorganisms to break down azo dyes into harmless products, is the most cost-effective and environmentally friendly strategy, as it requires less equipment and energy compared to other methods (12).

Microorganisms primarily decolorize dyes by using various enzymes to catalyze the cleavage of chemical bonds in azo dye molecules, thereby converting them into non-toxic or low-toxicity products. Enzymes involved in azo dye degradation mainly include azoreductases, laccases, and peroxidases (13). Among these, azoreductase is the key enzyme responsible for the reductive cleavage of azo bonds (14, 15). Under aerobic conditions, bacteria typically use specific azoreductases for decolorization. Although these enzymes themselves are generally not oxygen sensitive, oxygen competes with azo dyes for electrons within the electron transport chain, thereby reducing decolorization efficiency. Under anaerobic conditions, bacterial decolorization occurs via two mechanisms: (i) direct degradation of azo dyes by non-specific azoreductases and (ii) azo reduction mediated by redox mediators (16, 17). Anaerobic decolorization is highly efficient and represents the core pathway for dye degradation. However, the highly toxic aromatic amine intermediates are not readily further degraded under anaerobic conditions. Therefore, complete mineralization of azo dyes typically requires an anaerobic-aerobic combined process: during the anaerobic stage, azoreductases catalyze azo bond cleavage, converting dyes into aromatic amine intermediates; in the subsequent aerobic stage, oxidases such as laccases and peroxidases further degrade the aromatic amines into small molecules, ultimately mineralizing them to carbon dioxide and water (18).

*Shewanella* species are a group of gram-negative, facultative anaerobes widely distributed in marine, freshwater, and sedimentary environments, possessing a unique ability for extracellular electron transfer (19). Under anaerobic conditions, *Shewanella* can utilize various substances, including nitrate, fumarate, dimethyl sulfoxide, metal ions, and azo dyes, as terminal electron acceptors for anaerobic respiration (20). Many *Shewanella* strains have been shown to possess high azo dye decolorization capacity (21, 22). To date, the anaerobic decolorization mechanism of *Shewanella oneidensis* MR-1 is the best understood, with azo dye reduction primarily occurring through the Mtr pathway (23). In this pathway, the tetraheme c-type cytochrome CymA transfers electrons from menaquinone to downstream cytochromes, such as MtrA, MtrB, and MtrC, which ultimately deliver electrons to extracellular azo dyes (24–27). Additionally, *S. oneidensis* MR-1 can secrete flavins, melanin, and other redox mediators, which accelerate electron transfer to azo dyes (17, 28). While the core decolorization mechanism remains largely unchanged, there are species-specific differences in the exact composition of the pathway and the strategies employed. For instance, our previous studies revealed that *Shewanella putrefaciens* CN32 does not primarily depend on the Mtr pathway for azo dye reduction (29, 30). Another study also revealed that *Shewanella decolorationis* has a degradation pathway dominated by cytochrome b and flavin oxidoreductase, and proteins such as TorC and TorA play key roles in this pathway (31). Furthermore, the type of electron donor or dye can also influence the actual decolorization pathway (32, 33). Thus, the decolorization system of *Shewanella* is a multi-component network under precise regulation. A thorough understanding of its intracellular and extracellular electron transfer networks will reveal valuable targets for improvement and engineering applications.

In this work, we used mini-Tn5 insertions to identify genes in *S. putrefaciens* CN32 involved in the decolorization of acid yellow 36 (AY36) and investigated the mechanisms by which the target genes affect decolorization. Our results identified the menaquinone biosynthesis gene cluster in *S. putrefaciens* CN32 and revealed the critical role of menaquinone in both decolorization and biofilm formation.

## MATERIALS AND METHODS

### Construction of mini-Tn5 transposon insertion mutant library and initial screening for decolorization genes

Mini-Tn5 insertions were performed via triparental mating (34). In brief, the donor strain (*Escherichia coli* UQ3022/pRL27) and the helper strain were grown in LB medium containing the appropriate antibiotics. The strains were then harvested by centrifugation, washed with antibiotic-free LB medium, and mixed with a culture of *S. putrefaciens* CN32. The mixture was spotted onto LB plates. After incubating at 30°C for 8.5 h, the cells were collected and spread onto LB plates containing 50 µg/mL of kanamycin and 20 µg/mL of potassium tellurite. After 2 days of incubation at 30°C, black single colonies were selected and inoculated into 96-well plates containing 100 µL LB per well. After incubation at 30°C for 20 h, 1 µL of each culture was transferred to a new 96-well plate containing 100 µL of decolorization medium per well (yeast extract 2 g/L, NH_4_Cl 1 g/L, NaCl 0.5 g/L, Na_2_HPO_4_·12H_2_O 7.52 g/L, NaH_2_PO_4_·2H_2_O 7.13 g/L, sodium lactate 20 mM, and AY36 0.2 g/L). After 12 h of incubation at 30°C, the absorbance at 414 and 600 nm was measured. Strains exhibiting more than a 30% reduction in decolorization rate while maintaining growth similar to the wild-type were selected for further experiments.

### Localization of transposon insertion sites and in-frame gene deletion

Genomic DNA of *S. putrefaciens* CN32 was digested with restriction enzymes BamHI or SpeI, and the digested products were recovered and then self-circularized. The ligation products were transformed into *E. coli* UQ3021 and spread on LB plates containing appropriate antibiotics. Plasmids were extracted from purified colonies and subjected to sequencing analysis to identify the disrupted genes.

To create in-frame deletion mutants, the upstream and downstream homology arms of the target gene were amplified by PCR using *S. putrefaciens* CN32 genomic DNA as the template. The resulting fragments were fused together by PCR and then ligated into the suicide plasmid pK19*mobsacB*, creating the gene deletion vector. The vector was then transferred into *S. putrefaciens* CN32 via triparental mating, and single-crossover integrants were selected. Candidate clones were inoculated into LC medium (LB without NaCl) and then transferred onto LC plates containing 10% sucrose. The clones grown on these plates were subjected to colony PCR and sequencing to confirm the correct deletion of the target gene. The primers used in this study are listed in Table S1.

### Gene complementation and overexpression

Gene fragments containing the ribosome binding site and open reading frame were amplified by PCR from *S. putrefaciens* CN32 genomic DNA and ligated into pBBR1MCS-2 carrying a constitutive promoter. The resulting complementation plasmid was transferred into the deletion mutant and the wild-type strain via conjugation to create the complemented and overexpression strains, respectively.

### Decolorization and growth assays

An appropriate volume of *S. putrefaciens* CN32 seed culture was transferred into an anaerobic bottle containing 50 mL of decolorization medium to an initial OD_600_ of 0.1. After deoxygenation by sparging with sterile nitrogen, the bottles were immediately sealed and incubated statically at 30°C. Samples were withdrawn using a syringe to monitor growth and decolorization. Growth was assessed by measuring the absorbance at 600 nm. To determine the decolorization rate, the supernatant was collected by centrifugation, and its absorbance was measured at 414 nm. Decolorization rate (%) = (*A*_0_ – A_t_)/A_0_ ×100%, where A_0_ and A_t_ are the absorbances at 414 nm at the initial time and the sampling time, respectively.

### Biofilm formation assay

Biofilm formation by *S. putrefaciens* CN32 was measured following an established method (35). Briefly, 1% (vol/vol) of the *S. putrefaciens* CN32 seed culture was added to the decolorization medium, mixed, and transferred to 96-well plates (100 µL per well). After static incubation at 30°C, the absorbance at 600 nm was measured to assess growth. The medium was then discarded, and the wells were washed three times with deionized water. Subsequently, 150 µL of 1% (w/v) crystal violet solution was added to each well and incubated for 15 min for staining. After washing away excess crystal violet, 190 µL of 95% ethanol was added to each well to dissolve the crystal violet on the inner wall. Finally, the absorbance at 570 nm was measured to quantify biofilm formation.

### qRT-PCR

To examine target gene expression, total RNA of *S. putrefaciens* CN32 was extracted at the eighth hour of decolorization using the TRIzol method. After quantification, 2 µg of RNA was used for reverse transcription to obtain cDNA. qRT-PCR was then performed using SYBR Green with the cDNA as template. The qRT-PCR system and program are listed in Table S2 and S3. The 16S rRNA gene was used as an internal control.

### Determination of cytochrome c content

Cytochrome c content was determined using the QuantiChrom heme assay kit (BioAssay Systems, USA). Briefly, an appropriate amount of *S. putrefaciens* CN32 cells was lysed with B-PER reagent containing lysozyme. The lysate was centrifuged at 10,000 × *g* for 10 min at 4°C, and the supernatant was collected for protein concentration determination. The supernatant was appropriately diluted based on its protein concentration, and the diluted samples were transferred to a 96-well plate (50 µL per well). Then, 200 µL of the chromogenic reagent from the kit was added to each well. After 5 min of incubation, the absorbance at 405 nm was measured, and the cytochrome c concentration was calculated using a standard curve.

### Bioinformatics and statistical analysis

Gene function annotation was based on the NCBI and KEGG databases. Sequence alignments were performed using the DNAMAN v9 software package. All quantitative experiments were performed in triplicate. Significant differences between two groups were analyzed by *t*-test.

## RESULTS

### Screening and identification of decolorization genes

To identify genes involved in azo dye degradation, a mini-Tn5 transposon insertion mutant library of *S. putrefaciens* CN32 was constructed. A total of 1,000 insertion mutants were obtained and examined for their ability to decolorize AY36, a representative azo dye. Among them, four mutants, designated A-11B, F-4F, I-9C, and E-6E, showed markedly reduced decolorization capacity under anaerobic conditions compared to the wild type, while their growth rates were not significantly altered (Fig. S1). This suggested that genes involved in AY36 decolorization were disrupted in these four mutants. To identify the mini-Tn5 insertion sites, the genomic DNA of these mutants was digested, self-ligated, and transformed. Only positive transformants from A-11B and F-4F were successfully obtained. Sequencing analysis revealed that the disrupted gene in A-11B was *Sputcn32_0310*, and that in F-4F was *Sputcn32_1269*.

**Fig 1:**
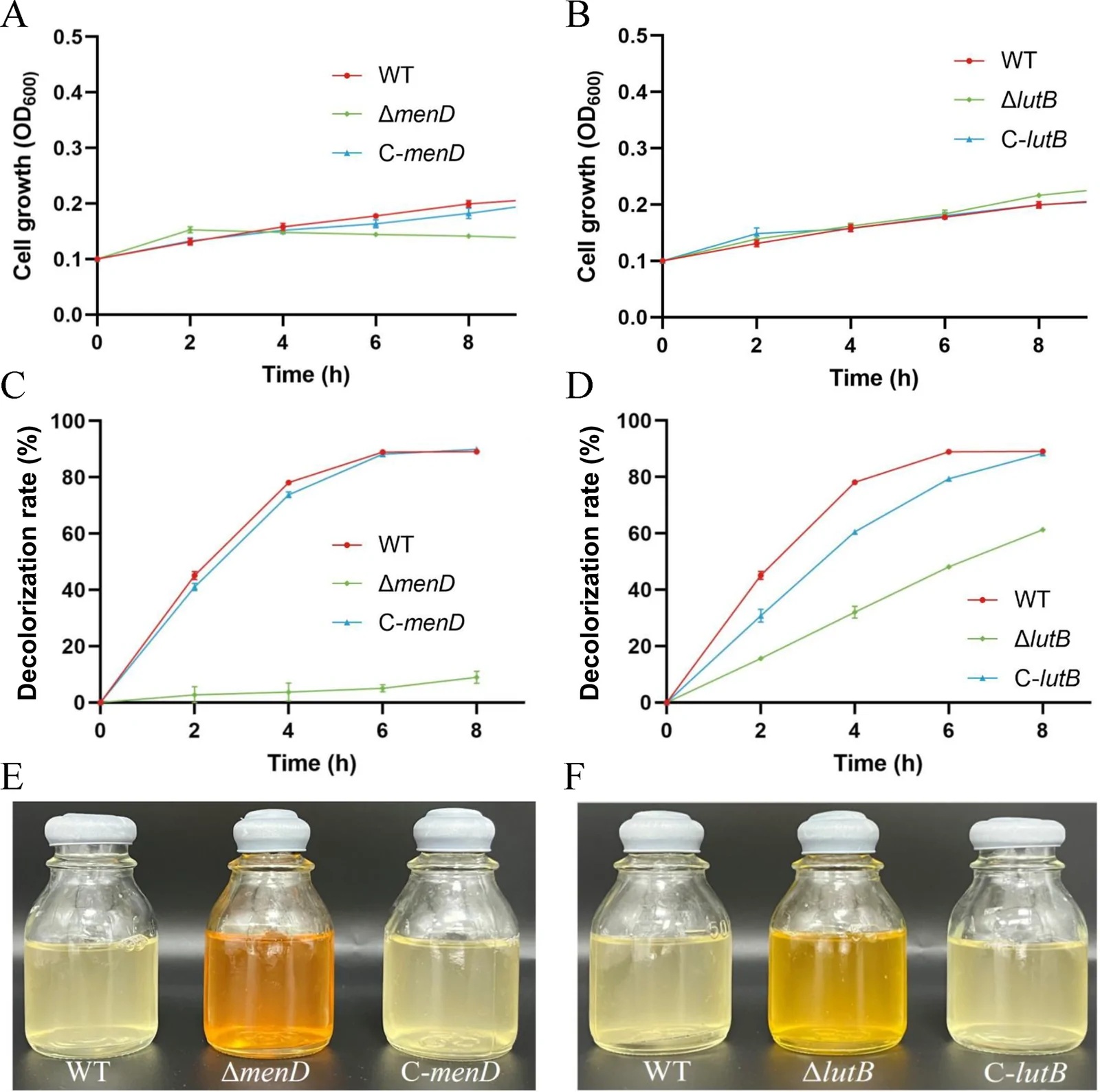
Decolorization rate and growth of mini-Tn5 insertion mutants. (A) Effect of mini-Tn5 insertion in the *menD* gene on cell growth. (B) Effect of mini-Tn5 insertion in the *lutB* gene on cell growth. (C) Effect of mini-Tn5 insertion in the *menD* gene on cell decolorization. (D) Effect of mini-Tn5 insertion in the *lutB* gene on cell decolorization. (E) Visualization of Δ*menD* decolorization. (F) Visualization of Δ*lutB* decolorization.

Sputcn32_0310 shares 91.27% sequence identity with MenD from the model strain *S. oneidensis* MR-1 (Fig. S2). MenD is 2-succinyl-5-enolpyruvyl-6-hydroxy-3-cyclohexene-1-carboxylic-acid synthase, a key enzyme in the bacterial menaquinone biosynthesis pathway. Sputcn32_1269 shares 91.81% sequence identity with LutB from *S. oneidensis* MR-1 (Fig. S3). LutB is a lactate utilization protein involved in lactate metabolism. To validate the roles of MenD and LutB in the anaerobic decolorization of AY36 by *S. putrefaciens* CN32, deletion mutants of each gene were constructed (Δ*menD* and Δ*lutB*). Both mutants had growth rates similar to the wild type but showed significantly lower decolorization rates (Fig. 1). For example, after 8 h of culture, the decolorization rate of the wild type was 89.1%, whereas those of Δ*menD* and Δ*lutB* were only 8.95% (*P* < 0.001) and 61.29% (*P* < 0.001), respectively. Complementation with *menD* or *lutB* restored decolorization (C-*menD* and C-*lutB*). These results indicate that *menD* and *lutB* are key genes responsible for anaerobic decolorization of AY36 in *S. putrefaciens* CN32. Since the loss of *menD* had a more pronounced negative effect on decolorization than the loss of lutB, subsequent studies focused on *menD*.

### Mechanism by which MenD affects decolorization

**Fig 2:**
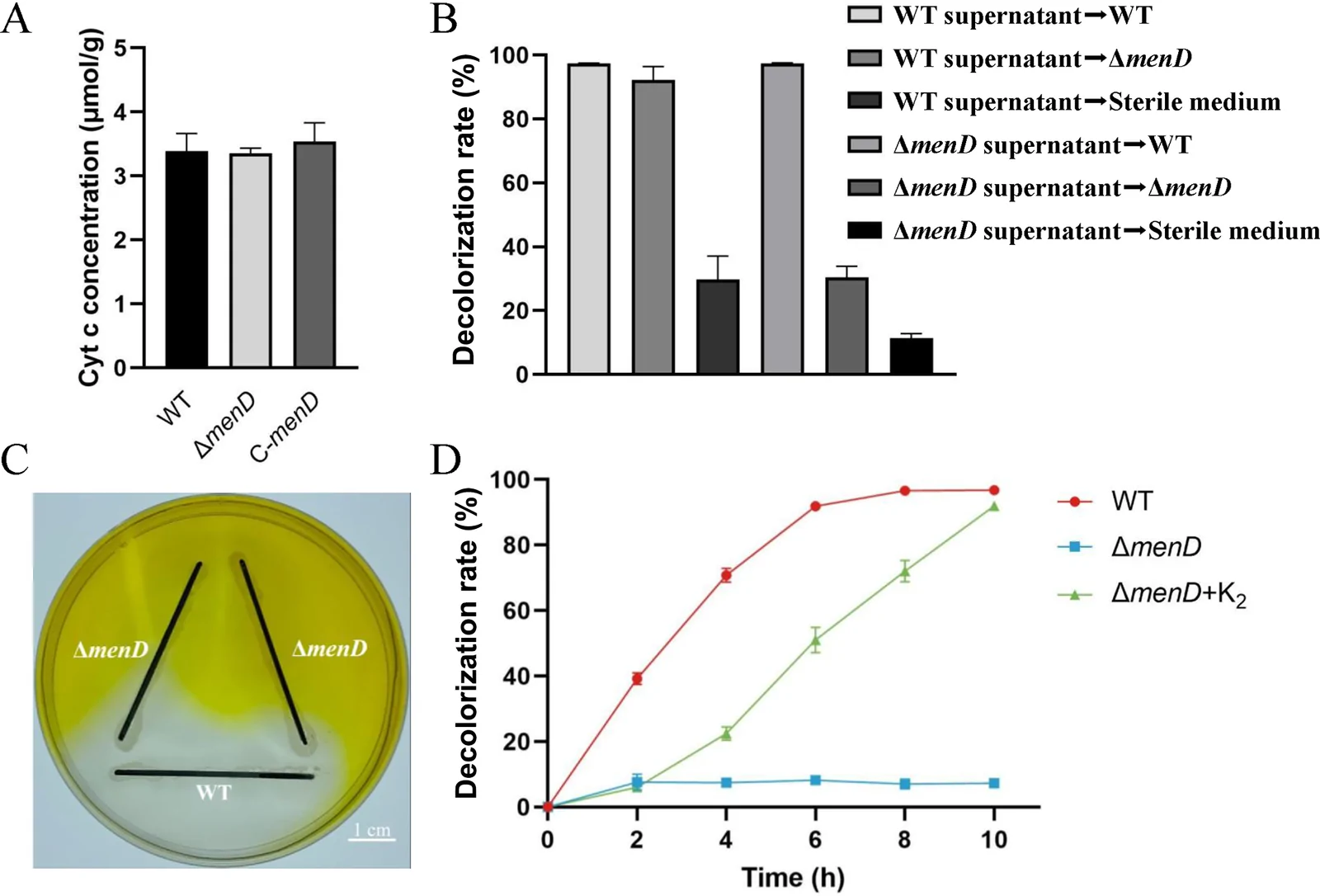
Effects of exogenous menaquinone on decolorization and growth of Δ*menD*. (**A**) Effect of *menD* deletion on intracellular cytochrome c content. (**B**) Effect of culture supernatant on decolorization efficiency. (**C**) Cross-feeding of Δ*menD* with wild-type on plates. (**D**) Effect of vitamin K_2_ supplementation on the decolorization efficiency of Δ*menD*. The medium supernatant and vitamin K_2_ solution were both sterilized by filtration before being added to the culture.

Anaerobic decolorization of azo dyes by *S. putrefaciens* CN32 is associated with electron transfer, in which cytochrome c is involved. Therefore, we measured the cytochrome c content of the wild type, Δ*menD*, and C-*menD*. No significant differences in cytochrome c content were observed among these strains (Fig. 2A), indicating that the reduced decolorization caused by *menD* deletion was not due to altered cytochrome c levels. Given that MenD is involved in menaquinone synthesis, we hypothesized that the defect in menaquinone synthesis in Δ*menD* impairs its decolorization capacity. To test this hypothesis, we performed supernatant complementation experiments. Adding the decolorization supernatant from the wild-type culture to the Δ*menD* mutant decolorization system restored (*P* < 0.01) the ability of Δ*menD* to decolorize AY36 (Fig. 2B). Cross-feeding experiments showed that Δ*menD* colonies grown adjacent to wild-type colonies on a plate could decolorize AY36 (Fig. 2C). These results suggest that the wild type produces an extracellular metabolite that enables Δ*menD* to accomplish anaerobic decolorization of the azo dye. Considering the role of MenD in menaquinone biosynthesis, we hypothesized that this extracellular metabolite is vitamin K_2_ (menaquinone) or a precursor of it. To test this, chemical complementation experiments were performed, where supplementation of the Δ*menD* mutant decolorization medium with 0.05 mM vitamin K_2_ significantly improved (*P* < 0.01) decolorization efficiency (Fig. 2D). These experiments demonstrate that menaquinone is essential for anaerobic decolorization of AY36 by *S. putrefaciens* CN32.

### Role of menaquinone biosynthesis genes in decolorization

Since deletion of *menD* severely impaired the decolorization capacity of *S. putrefaciens* CN32, we hypothesized that overexpression of *menD* would enhance decolorization. To test this, we constructed a *menD* overexpression strain (O-*menD*). Contrary to expectations, O-*menD* did not show improved decolorization compared to the wild type (Fig. S4). This may be because menaquinone biosynthesis requires multiple enzymes, and overexpressing *menD* alone is insufficient to boost decolorization.

**Fig 3:**
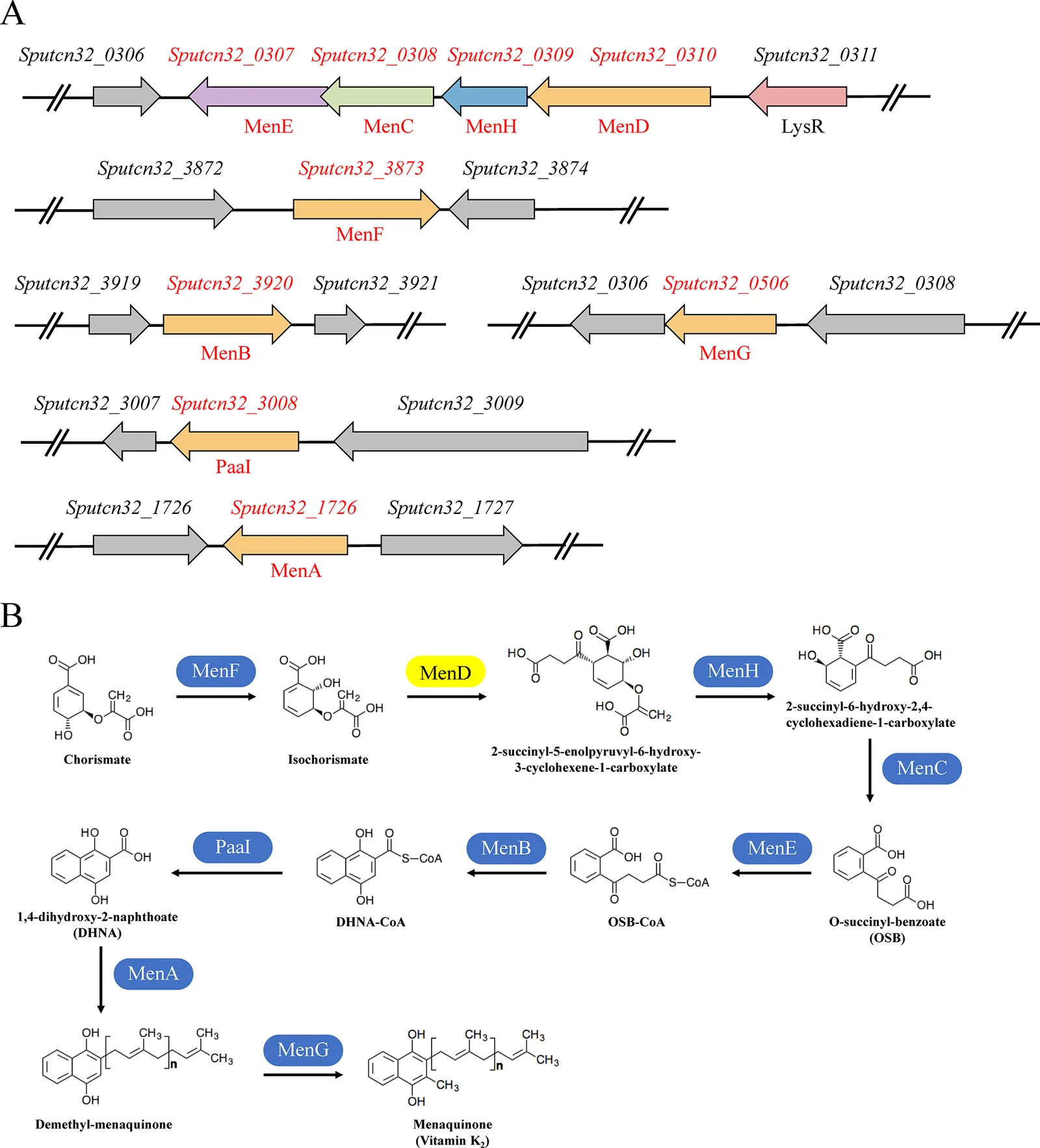
(**A**) Menaquinone synthesis genes and (**B**) synthesis pathway in *S. putrefaciens* CN32.

**Fig 4:**
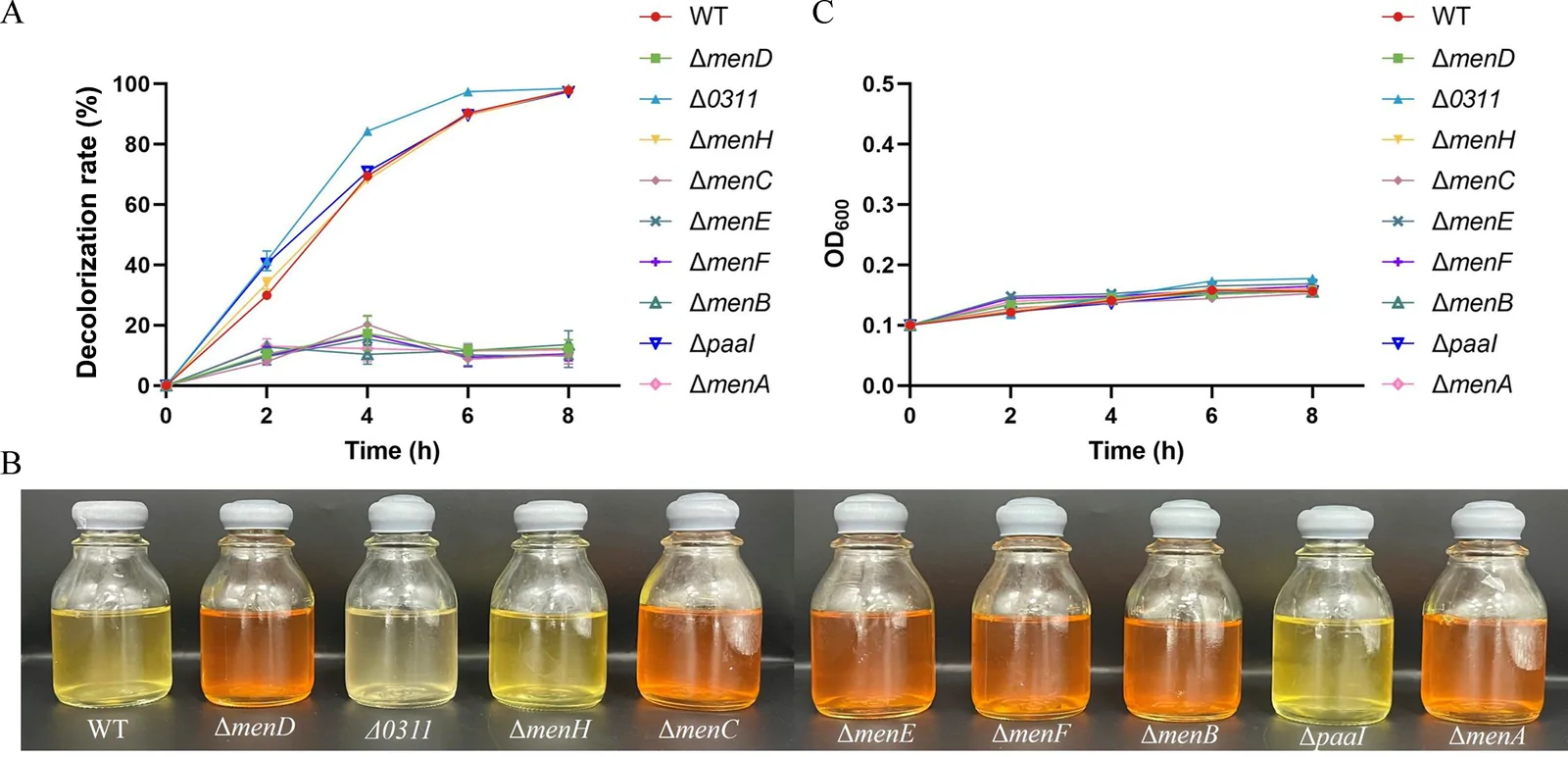
Decolorization and growth of single-deletion mutants of menaquinone synthesis genes. (A) Effect of single-gene deletion on decolorization. (B) Visualization of decolorization by the mutant strains. (C) Effect of single-gene deletion on growth.

In the model strain *S. oneidensis* MR-1, all genes encoding enzymes involved in menaquinone biosynthesis have been identified. However, in *S. putrefaciens* CN32, the full set of relevant genes has not yet been described. By analyzing the upstream and downstream sequences of *menD* and performing sequence alignments, we identified the genes encoding key enzymes involved in menaquinone biosynthesis in *S. putrefaciens* CN32 (Fig. 3). In addition to *menD*, eight other genes in the menaquinone biosynthesis pathway were identified: *menA*, *menB*, *menC*, *menE*, *menF*, *menG*, *menH*, and *paaI* (Table S4). Furthermore, a LysR-family transcriptional regulator encoded by *Sputcn32_0311* was found adjacent to the *men* gene cluster. Reverse transcription PCR confirmed that *Sputcn32_0311* and four *men* genes (*menC*, *menD*, *menE*, and *menH*) are co-transcribed in the same operon (Fig. S5). Thus, 0311 may affect menaquinone synthesis by regulating transcription of its gene cluster. To investigate whether these genes are involved in azo dye decolorization, single-deletion mutants of each gene were constructed and tested for their ability to decolorize AY36. The results showed that deletion of any gene in the biosynthesis pathway, with the exception of *menH*, *paaI*, and *Sputcn32_0311*, led to a marked decrease (*P* < 0.001) in decolorization capacity (Fig. 4). This suggests that the reduction of AY36 is highly dependent on an intact menaquinone biosynthesis pathway.

### Effect of menaquinone synthesis genes on AY36-induced biofilm formation in *S. putrefaciens* CN32

**Fig 5:**
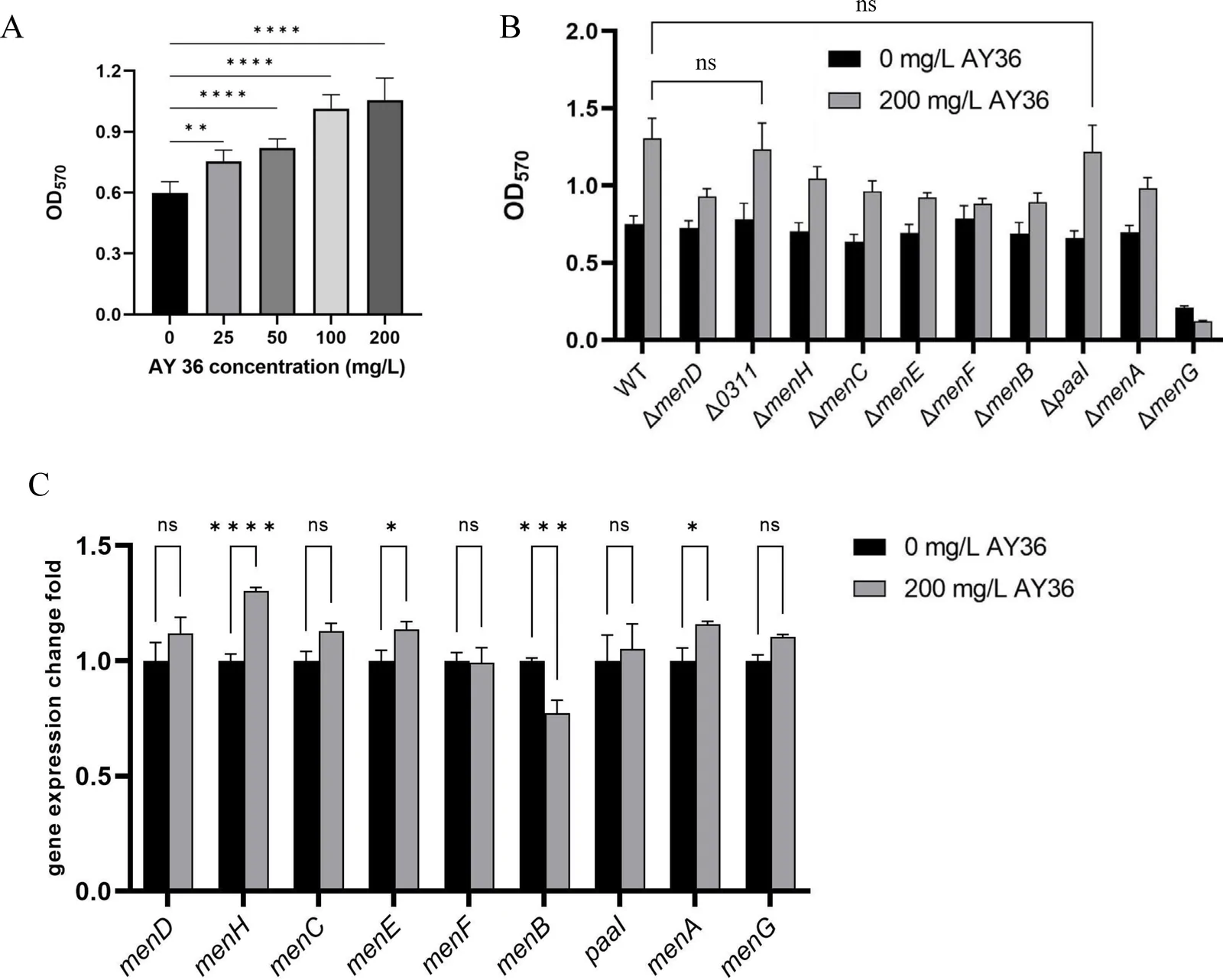
Effect of AY36 on biofilm formation in *S. putrefaciens* CN32. (**A**) Effect of AY36 concentration on biofilm formation in the wild type. (**B**) Effect of AY36 on biofilm formation in menaquinone synthesis gene mutants. (**C**) Effect of AY36 on transcription of menaquinone synthesis genes. *P < 0.05, **P < 0.01, ***P < 0.001, ****P <0.0001 and ns, not signfance.

Since electron transfer can affect biofilm formation, we investigated the impact of AY36 on biofilm formation in wild type and menaquinone synthesis-deficient mutants. The addition of AY36 in the range of 25–200 mg/L was positively correlated with biofilm formation and had no significant effect on the growth of *S. putrefaciens* CN32 (Fig. 5A). In addition, supplementation with 200 mg/L sodium chloride or glucose did not enhance biofilm formation, indicating that AY36 acts specifically as an azo dye to induce biofilm formation rather than through osmotic effects (Fig. S6). To investigate the role of menaquinone synthesis genes in this induction, we measured biofilm formation in wild type and mutant strains in the presence of 200 mg/L AY36. The results showed that, except for *Sputcn32_0311* and *paaI*, deletion of any gene in the menaquinone biosynthesis pathway resulted in significantly reduced biofilm formation compared to the wild type (Fig. 5B). These results indicate that menaquinone plays an important role not only in decolorization but also in AY36-induced biofilm formation. To further investigate whether AY36 induces the expression of menaquinone synthesis genes, we examined the expression levels of these genes in the wild type using qRT-PCR. The results showed that AY36 had no significant effect on the expression of five out of the nine genes in the *men* gene cluster (Fig. 5C). The largest change was observed for *menH*, with an upregulation of only 30%. These results suggest that AY36 does not induce biofilm formation by upregulating the transcription of genes related to menaquinone synthesis. In summary, AY36 significantly induces *S. putrefaciens* CN32 biofilm formation in a dose-dependent manner, and an intact menaquinone biosynthesis pathway enhances this effect.

## DISCUSSION

**Fig 6:**
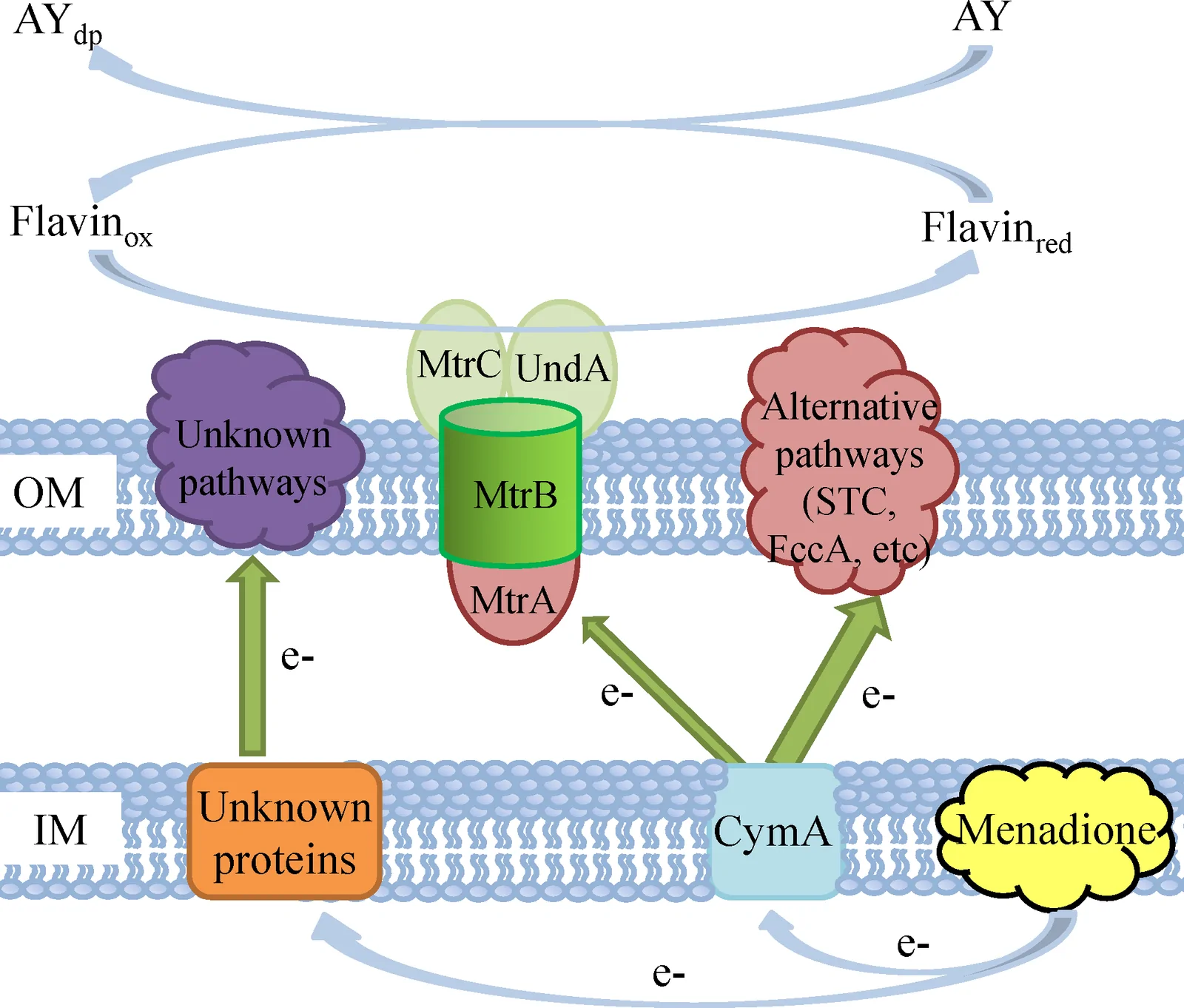
A proposed molecular model of AY36 decolorization in *S. putrefaciens* CN32.

In this study, we identified two genes in *S. putrefaciens* CN32 involved in AY36 decolorization using mini-Tn5 insertion. Their encoded proteins, MenD and LutB, are involved in menaquinone synthesis and lactate metabolism, respectively, indicating that anaerobic decolorization is related to respiratory chain cofactors and electron donors. The Mtr pathway has long been considered the primary decolorization route in *Shewanella*. In this pathway, CymA transfers electrons from the quinone pool to MtrA; subsequently, outer membrane multi-heme cytochromes (MtrC, OmcA, etc.) accept electrons from MtrA to reduce dyes (36–38). In addition, alternative pathways exist. For example, CymA can transfer electrons to other periplasmic proteins such as STC and FccA instead of MtrA (39–41). In all these pathways, CymA plays a central role. Previous studies have shown that the Δ*cymA* mutant retains a decolorization rate of over 40% at the end of the culture period, while the present study found that the Δ*menD* mutant loses almost all of its decolorization capacity (less than 10%) (29). This suggests that in *S. putrefaciens* CN32, the contribution of menaquinone to decolorization goes beyond its role through CymA and that there exists a CymA-independent electron transfer branch (Fig. 6). Similar results have been reported in *S. oneidensis* MR-1, where certain proteins can partially compensate for CymA function when it is impaired (42). For example, in a *cymA*-deletion mutant of *S. oneidensis* MR-1, cells activate an alternative electron transfer pathway based on SirCD, which transfers electrons from the quinone pool to various terminal oxidoreductases (43). Additionally, when the electron acceptor reduction rate is low, *S. oneidensis* MR-1 activates alternative CymA-independent pathways for electron transfer (44). These findings highlight the complexity of the electron transfer network in *Shewanella*, and the role of non-CymA-dependent pathways in azo dye reduction warrants further investigation.

By sequence alignment with the corresponding proteins from *S. oneidensis* MR-1, we identified the genes encoding all enzymes involved in menaquinone biosynthesis in *S. putrefaciens* CN32. Deletion of most of these genes abolished decolorization, confirming their essential roles in menaquinone synthesis. *menH* and *paaI* were exceptions. Deletion of these genes did not significantly affect decolorization compared to the wild type, suggesting the possible existence of salvage pathways or isozymes that maintain normal menaquinone synthesis and electron transfer. Several studies support this speculation. For example, in some Bacteroidetes, the thioesterase function encoded by *paaI* can be replaced by fusion proteins and homologous thioesterases (45). Moreover, *men* mutants of *S. oneidensis* MR-1 can restore menaquinone synthesis by taking up a 1,4-dihydroxy-2-naphthoic acid derivative secreted by the wild type (46, 47). *Sputcn32_0311* encodes a LysR-family transcriptional regulator that is co-transcribed with four *men* family genes in the same operon. However, deletion of this gene did not affect decolorization in *S. putrefaciens* CN32, suggesting that the decolorization process is not regulated by this gene under the tested conditions. The regulatory network under azo dye stress is highly complex, with global regulators such as RpoS and Crp playing key roles (48). The function of 0311 requires further exploration under other experimental conditions.

Most menaquinone synthesis gene deletion mutants showed significantly reduced biofilm formation in the presence of the azo dye, while Δ*menH* and Δ*paaI*, whose decolorization rate was similar to the wild type, exhibited normal biofilm formation. This suggests that the integrity of the electron transfer chain plays a crucial role in biofilm formation induced by the azo dye AY36. Previous studies have established that bacterial electron transfer capacity is closely linked to biofilm formation. For example, Hernández-Eligio et al. showed that the transcription factor CsrA in *Geobacter sulfurreducens* simultaneously regulates genes involved in extracellular electron transfer and biofilm formation; deletion of *csrA* affects both current production and biofilm formation (49). The electron transfer chain may influence biofilm formation by directly altering cellular energy metabolism and membrane potential. Qin et al. demonstrated that in *Bacillus subtilis*, the function of the electron transfer chain directly affects membrane potential establishment, which is a key factor in biofilm matrix synthesis (50). Our qRT-PCR results showed that AY36 did not significantly upregulate transcription of the *men* gene cluster, suggesting that the azo dye induces biofilm formation through direct stimulation rather than by enhancing menaquinone synthesis. A deficiency in menaquinone synthesis impairs electron transfer, resulting in an inability to form a high-level biofilm even under dye stress. This leads to significantly reduced biofilm formation in the deletion mutants.

### Conclusion

This study reveals the critical role of menaquinone in the anaerobic decolorization of azo dyes by *S. putrefaciens* CN32, systematically identifies the genes involved in the menaquinone biosynthesis pathway, and elucidates the importance of an intact electron transfer chain for both decolorization capacity and biofilm formation. These findings provide a theoretical foundation for a deeper understanding of the decolorization mechanism and for optimizing wastewater treatment processes.

## Data Availability

The data sets used and analyzed during the current study are available from the corresponding author upon reasonable request.

## SUPPLEMENTAL MATERIAL

Figure S1 spectrum.01996-26-s0001.tif Decolorization capacity of mini‑Tn5 insertion mutants.

Figure S2 spectrum.01996-26-s0002.tif Sequence alignment of Sputcn32_0310 from *S*. putrefaciens CN32 with MenD from *S*. oneidensis MR-1.

Figure S3 spectrum.01996-26-s0003.tif Sequence alignment of Sputcn32_1269 from *S*. putrefaciens CN32 with LutB from *S*. oneidensis MR-1.

Figure S4 spectrum.01996-26-s0004.tif Effect of menD overexpression on decolorization.

Figure S5 spectrum.01996-26-s0005.pdf Co-transcription analysis of Sputcn32_0311 with menD, menH, menC, and menE.

Figure S6 spectrum.01996-26-s0006.tif Effects of sodium chloride and glucose on biofilm formation of *S. putrefaciens CN32*.

Tables S1 to S4 spectrum.01996-26-s0007.docx The primers, qRT-PCR system, and protein sequences involved in this study.

Supplemental legends spectrum.01996-26-s0008.docx Legends for Fig. S1 to S6.

ASM does not own the copyrights to Supplemental Material that may be linked to, or accessed through, an article. The authors have granted ASM a non-exclusive, world-wide license to publish the Supplemental Material files. Please contact the corresponding author directly for reuse.
