# Supplementary material for: Menaquinone synthesis genes are critical for the decolorization of acid yellow 36 in Shewanella putrefaciens CN32
Source: Microbiol Spectr. 2026 Sep 2;14(10):e01996-26. doi: 10.1128/spectrum.01996-26 (PMC13688807; doi:10.1128/spectrum.01996-26)
Supplement: Supplementary file 1 [file spectrum.01996-26-s0005.pdf]

A

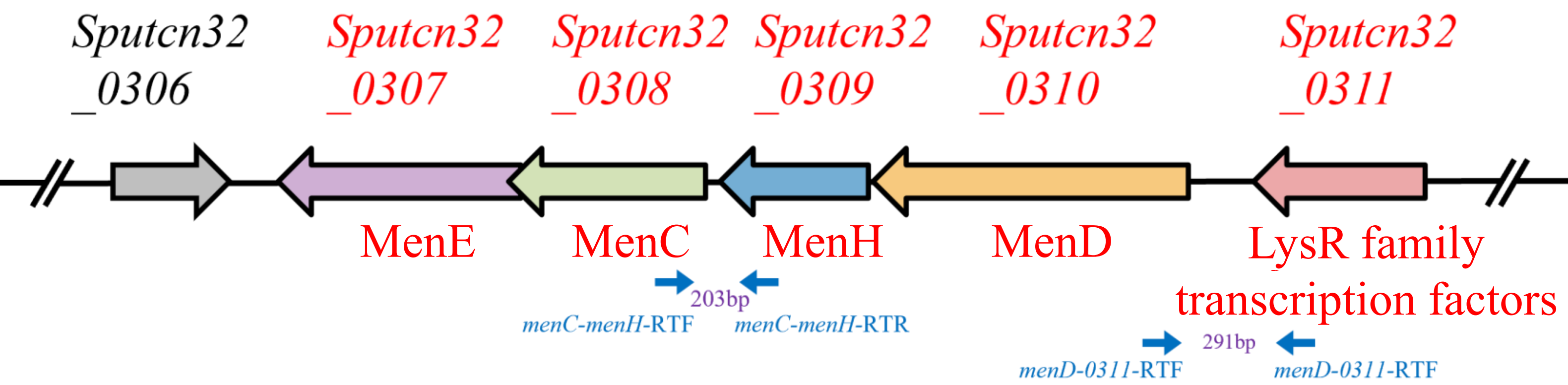
Sputcn32 _0306
Sputcn32 _0307
Sputcn32 _0308
Sputcn32 _0309
Sputcn32 _0310
Sputcn32 _0311
MenE
MenC
MenH
MenD
LysR family transcription factors
203bp
menC-menH-RTF
menC-menH-RTR
291bp
menD-0311-RTF
menD-0311-RTF

B

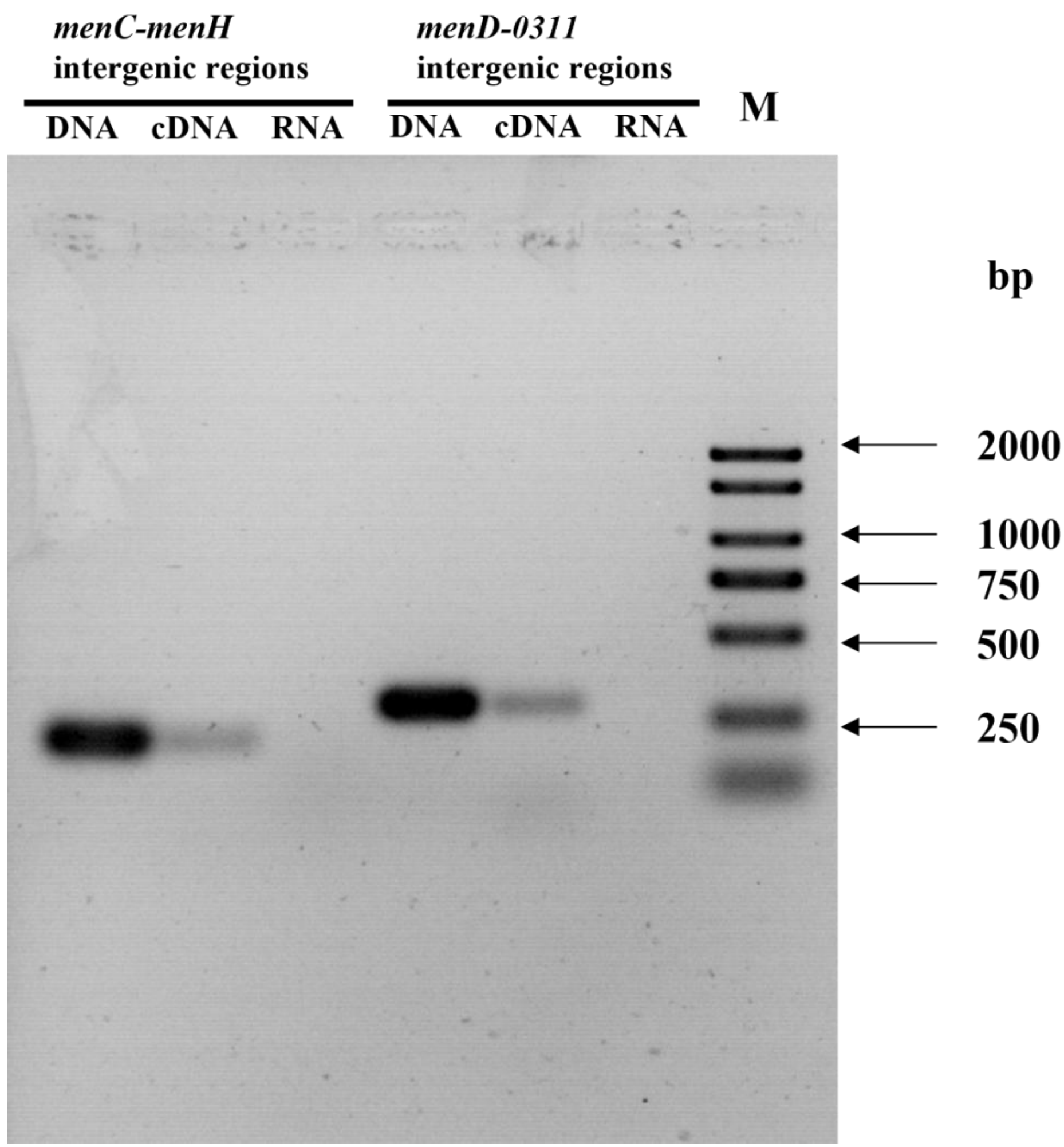
menC-menH intergenic regions
menD-0311 intergenic regions
DNA
cDNA
RNA
DNA
cDNA
RNA
M
bp
2000
1000
750
500
250
