# Supplementary material for: Menaquinone synthesis genes are critical for the decolorization of acid yellow 36 in Shewanella putrefaciens CN32
Source: Microbiol Spectr. 2026 Sep 2;14(10):e01996-26. doi: 10.1128/spectrum.01996-26 (PMC13688807; doi:10.1128/spectrum.01996-26)
Supplement: Supplementary file 2 [file spectrum.01996-26-s0007.docx]

**Table S1 The primers used in this study**

| **Name** | **Sequence** | **Function** |
| --- | --- | --- |
| 0310-D5F-EcoRI | AGCGGGGAATTCTGCCATTTGGTGTATGTTG | *Sputcn32_0310* knockout |
| 0310-D5R-BamHI | ATCTTCGGATCCAATCTGCTGCCTGTTCCT | *Sputcn32_0310* knockout |
| 0310-D3F-BamHI | GCCCCAGGATCCAATAAGATTGAGTGTGGCTGTATT | *Sputcn32_0310* knockout |
| 0310-D3R-PstI | GCTACTGCTGCAGCATTTATATCCAATTGCAAAAGA | *Sputcn32_0310* knockout |
| 0310-UF | GTTTCACCCATAAGTTGAGCT | *Sputcn32_0310* knockout |
| 0310-DR | CGTTACACTAAGCCCATCTTTA | *Sputcn32_0310* knockout |
| 0310-OF | TATCGTGCTGCTCAATATCAA | *Sputcn32_0310* knockout |
| 0310-OR | GCCTAAAATCAGTCGTAGCCTTAA | *Sputcn32_0310* knockout |
| 0310-InF | TCTAGTGGACGAGTATTTGCA | *Sputcn32_0310* knockout |
| 0310-InR | CATCGGAAATATTGACCAACT | *Sputcn32_0310* knockout |
| 0310-CF | GTAAACAGATCTGCGTTACACTAAGCCCATC | *Sputcn32_0310* complementation |
| 0310-CR | CGTAACGAAATTCGACCGGTAGTGTAGTTTCAC | *Sputcn32_0310* complementation |
| 1269-D5F-EcoRI | AGGACGGAATTCATCCACAGACTGACTTGAAC | *Sputcn32_1269* knockout |
| 1269-D5R-SpeI | TGGGGCACTAGTAAATACCGTGAACTCCCAGT | *Sputcn32_1269* knockout |
| 1269-D3F-SpeI | GTGGAGACTAGTTTTAATTTCTGAACCAAGCTGA | *Sputcn32_1269* knockout |
| 1269-D3R-PstI | ACTTATCTGCAGTTCGCTAAGATAGGCCTATATTT | *Sputcn32_1269* knockout |
| 1269-UF | CGATAAGTTCATCTACTTTTGCTT | *Sputcn32_1269* knockout |
| 1269-DR | AAGCAGATATATTTTGTCGCGATG | *Sputcn32_1269* knockout |
| 1269-OF | TAATACCTCGTTTCATTTACTCACC | *Sputcn32_1269* knockout |
| 1269-OR | AGCCGATAGTCAATGAGTTAATAT | *Sputcn32_1269* knockout |
| 1269-InF | GTCGACGATAATAATGTGCATC | *Sputcn32_1269* knockout |
| 1269-InR | ATTTAGGTGAGCGCATTATTCA | *Sputcn32_1269* knockout |
| 1269-CF | ATTGCCGGATCCGATTGAAATTCGCCACATAGC | *Sputcn32_1269* complementation |
| 1269-CR | TTACTAGAATTCGACATCTTATAGGCTCCTGTG | *Sputcn32_1269* complementation |
| 0311-D5F-EcoRI | AAGAAGGAATTCGAGTCAGGGTTCGGATAAG | *Sputcn32_0311* knockout |
| 0311-D5R-BamHI | TTCGTCGGATCCACCAAAAATTTCCCGTAGA | *Sputcn32_0311* knockout |
| 0311-D3F-BamHI | AATGCCGGATCCATCAATGAGGATCTAAAATGGT | *Sputcn32_0311* knockout |
| 0311-D3R-PstI | TGAGCTCTGCAGGTTGTGGATCTTGTTCAGG | *Sputcn32_0311* knockout |
| 0311-UF | GAAGGCTCTTGATATTCCAG | *Sputcn32_0311* knockout |
| 0311-DR | GTCCGCATAACTGAATCTATTA | *Sputcn32_0311* knockout |
| 0311-OF | AGTCCTCACATTTATCCGTAG | *Sputcn32_0311* knockout |
| 0311-OR | TAATAGTTGGTCAATATTTCCG | *Sputcn32_0311* knockout |
| 0311-InF | CTCATTTATATCCAATTGCAAA | *Sputcn32_0311* knockout |
| 0311-InR | AATATCGCTTACCATTTCGTA | *Sputcn32_0311* knockout |
| 0309-D5F-EcoRI | GCTGCCGAATTCATCGGAAATATTGACCAACT | *Sputcn32_0309* knockout |
| 0309-D5R-BamHI | CAGATCGGATCCCACACAAATGCAATGAAAG | *Sputcn32_0309* knockout |
| 0309-D3F-BamHI | GCACCCGGATCCGCTGCATTAATCTCAAAAATC | *Sputcn32_0309* knockout |
| 0309-D3R-PstI | CAGGATCTGCAGCATTTAGCGATTCATCCAA | *Sputcn32_0309* knockout |
| 0309-UF | ACGGCTTAGAATTTGGTTAC | *Sputcn32_0309* knockout |
| 0309-DR | AGTGGCAGTCGATATTGATAC | *Sputcn32_0309* knockout |
| 0309-OF | ACTCAATACGGTAAATATAAGCAG | *Sputcn32_0309* knockout |
| 0309-OR | GCGATATCGATTAACTGAGATA | *Sputcn32_0309* knockout |
| 0309-INF | GATAATCAAGACGAGTTAGCG | *Sputcn32_0309* knockout |
| 0309-INR | AAATTTACTATCTTCAGTGCCC | *Sputcn32_0309* knockout |
| 0308-D5F-PstI | AAACGCCTGCAGAAGCCAGTGACCAAATTG | *Sputcn32_0308* knockout |
| 0308-D5R-BamHI | AATATCGGATCCGTGCTGCTCAATATCAATACC | *Sputcn32_0308* knockout |
| 0308-D3F-BamHI | CAAGATGGATCCTTAATTGTGTCATCGGGTAA | *Sputcn32_0308* knockout |
| 0308-D3R-EcoRI | CCGATAGAATTCTGAAATAACGGTAGAGAGAGTAAG | *Sputcn32_0308* knockout |
| 0308-UF | CTGCCACTTGAGGTATTACTC | *Sputcn32_0308* knockout |
| 0308-DR | CGTCTGATAATTGATGCTTTT | *Sputcn32_0308* knockout |
| 0308-OF | TAGATTTTCAAGGGGCTTC | *Sputcn32_0308* knockout |
| 0308-OR | GAGTTTATCACGCGTTATCTG | *Sputcn32_0308* knockout |
| 0308-INF | GCAACACATCGATAAACTTATC | *Sputcn32_0308* knockout |
| 0308-INR | CCATAAATTAAACTTAGCTCATCTT | *Sputcn32_0308* knockout |
| 0307-D5F-EcoRI | TGCTTGGAATTCCCATTAGAGGCTATCGAATATAT | *Sputcn32_0307* knockout |
| 0307-D5R-XbalI | ATGCGTTCTAGACTGATAATTGATGCTTTTTCC | *Sputcn32_0307* knockout |
| 0307-D3F-XbalI | CAAATGTCTAGAGGGCTAAAGGTGAATAGAAA | *Sputcn32_0307* knockout |
| 0307-D3R-PstI | GAAGACTGCAGAAAATTAATTGTGCAGCAGTT | *Sputcn32_0307* knockout |
| 0307-UF | TTAATTGTGTCATCGGGTAA | *Sputcn32_0307* knockout |
| 0307-DR | AGCCTTTAATAACGGTTGG | *Sputcn32_0307* knockout |
| 0307-OF | CGAAGATGAGCTAAGTTTAATTT | *Sputcn32_0307* knockout |
| 0307-OR | TCAATATTGTGACCACTAATCAG | *Sputcn32_0307* knockout |
| 0307-INF | CCTTGAAATGATCTGCCTATA | *Sputcn32_0307* knockout |
| 0307-INR | GTCAATGAGAGGATGTTGTTT | *Sputcn32_0307* knockout |
| 3873-D5F-EcoRI | ACCTGAGAATTCTGACCTGATGACCTGATAGA | *Sputcn32_3873* knockout |
| 3873-D5R-BamHI | AATTAAGGATCCAGGCATAGAGACAGTTGTTATG | *Sputcn32_3873* knockout |
| 3873-D3F-BamHI | GCCGAAGGATCCTGGCAGGAACTTGAGAATA | *Sputcn32_3873* knockout |
| 3873-D3R-PstI | CCTGCACTGCAGTCACAATGGCAACTTAATGT | *Sputcn32_3873* knockout |
| 3873-UF | AGGGCTTATATCTTGACACCT | *Sputcn32_3873* knockout |
| 3873-DR | CTTAAACAGCATTTTGATCGT | *Sputcn32_3873* knockout |
| 3873-OF | TTAAACCTGCCAATTGATATC | *Sputcn32_3873* knockout |
| 3873-OR | TGCCATATTTTACTTATGCTTG | *Sputcn32_3873* knockout |
| 3873-INF | GATTTGAAACAGGTGTCGA | *Sputcn32_3873* knockout |
| 3873-INR | AGGAAATAAAGGTGCGTTC | *Sputcn32_3873* knockout |
| 3920-D5F-EcoRI | AATTAAGAATTCTGGTTATTCACTTATGTTGGAC | *Sputcn32_3920* knockout |
| 3920-D5R-BamHI | TGCATGGGATCCGTCGAGTGCAATGTAAAGC | *Sputcn32_3920* knockout |
| 3920-D3F-BamHI | GCCTTCGGATCCCCTTGGCACTACTAATTTTAGAA | *Sputcn32_3920* knockout |
| 3920-D3R-PstI | AGTGTGCTGCAGATGAGATAACTGGTTTAGAGCTTT | *Sputcn32_3920* knockout |
| 3920-UF | TCGATCAACAATTAGCAAAA | *Sputcn32_3920* knockout |
| 3920-DR | GACAAAATGGCGATTAACTTA | *Sputcn32_3920* knockout |
| 3920-OF | CATCTGGCTGGTGAATCT | *Sputcn32_3920* knockout |
| 3920-OR | CTATGTCAAACTTCCATTTTAGG | *Sputcn32_3920* knockout |
| 3920-InF | GCAAAGATGGCTATAAATATGA | *Sputcn32_3920* knockout |
| 3920-InR | GCACTGTAGTTAAAACCGC | *Sputcn32_3920* knockout |
| 3008-D5F-PstI | GACTTACTGCAGCTCTTATAGTTACAATGGCGAA | *Sputcn32_3008* knockout |
| 3008-D5R-BamHI | ACCGATGGATCCTTCACTTATCTCAATCTCTAGGG | *Sputcn32_3008* knockout |
| 3008-D3F-BamHI | ATTCACGGATCCAACAGTGCAGGTGAGTTAACT | *Sputcn32_3008* knockout |
| 3008-D3R-EcoRI | AGGATTGAATTCAAACACAACGAAGGCAAG | *Sputcn32_3008* knockout |
| 3008-UF | TGTGCTTCTGAATGCTTTC | *Sputcn32_3008* knockout |
| 3008-DR | CAACCGATGCTAGATAACCTA | *Sputcn32_3008* knockout |
| 3008-OF | AGGGCTATGGTCTTATCAACT | *Sputcn32_3008* knockout |
| 3008-OR | CGCTGTGTCATAGATAAAACTC | *Sputcn32_3008* knockout |
| 3008-InF | TCGGTGATGACTACTTAAAGG | *Sputcn32_3008* knockout |
| 3008-InR | TAACAATCCATTACGAGAAGC | *Sputcn32_3008* knockout |
| 1726-D5F-EcoRI | CAGCACGAATTCAGTGAACTGTAGGTTGATCGA | *Sputcn32_1726* knockout |
| 1726-D5R-BamHI | GAGCATGGATCCGGAGGTTGCAGCAATTAA | *Sputcn32_1726* knockout |
| 1726-D3F-BamHI | AGTACCGGATCCTTGTTTAGCGTGGGTTTAG | *Sputcn32_1726* knockout |
| 1726-D3R-PstI | ACTTAACTGCAGGTTGATGTATCCTGAAGCG | *Sputcn32_1726* knockout |
| 1726-UF | GCGACAAATATCATTCCATAT | *Sputcn32_1726* knockout |
| 1726-DR | ATGCCGAACGTAACTTTAAT | *Sputcn32_1726* knockout |
| 1726-OF | ACATCGAGGACAAACTGG | *Sputcn32_1726* knockout |
| 1726-OR | CAGAATTAGCCGAACAGATT | *Sputcn32_1726* knockout |
| 1726-InF | CTTGCTAACGACTACTTTGATTT | *Sputcn32_1726* knockout |
| 1726-InR | AAAGACAAATGCCGCTAC | *Sputcn32_1726* knockout |
| 0506-D5F-EcoRI | GTCGACGAATTCGCTATCGCTAAAATGCCTAG | *Sputcn32_0506* knockout |
| 0506-D5R-BamHI | CTCAATGGATCCGGTATGACGCTTCCAGAA | *Sputcn32_0506* knockout |
| 0506-D3F-BamHI | GTCGCAGGATCCTTGCACCGTGGTTATAAGT | *Sputcn32_0506* knockout |
| 0506-D3R-PstI | GGTCATCTGCAGGGCTTAATGTGGTCTCTTAATT | *Sputcn32_0506* knockout |
| 0506-UF | GTAAACAAAGACACTGACGTTAGT | *Sputcn32_0506* knockout |
| 0506-DR | GTGGCTGAGTACTTGGATCT | *Sputcn32_0506* knockout |
| 0506-OF | AGCTTGCAACAGATTTGG | *Sputcn32_0506* knockout |
| 0506-OR | TTAATATAAACAGGGCCGAG | *Sputcn32_0506* knockout |
| 0506-InF | CGATTTAACTGCAAAATTCTC | *Sputcn32_0506* knockout |
| 0506-InR | GGTAATAATGTCGAAGTGGTTAT | *Sputcn32_0506* knockout |
| 0310-QF | GACATGTATGCCCCAGTCAGTTGC | qRT-PCR |
| 0310-QR | CAAGCCGTTGCCAGTAAACCGTCA | qRT-PCR |
| 0309-QF | CTATTCTCTCGGTGGCCGTATTGC | qRT-PCR |
| 0309-QR | CCCGGATGACAGGATTCGAGGGTT | qRT-PCR |
| 0308-QF | TGAGATGTATGAGGGACTCGCGGC | qRT-PCR |
| 0308-QR | GCTTAAAATGCAACGCACGCCATG | qRT-PCR |
| 0307-QF | CCGCCATATCCCCCTTACACCAAG | qRT-PCR |
| 0307-QR | TCACCAAGGGCGATAACCTGTTGG | qRT-PCR |
| 3873-QF | GCAAATGCCCTGCTCGAAGACAAT | qRT-PCR |
| 3873-QR | CATAATGGCTCAACGGCGTGAGCA | qRT-PCR |
| 3920-QF | CTTTTCTTCCGGTGGCGATCAACG | qRT-PCR |
| 3920-QR | ATTCTGGCTTGATCGGCTTTACCT | qRT-PCR |
| 3008-QF | CACCCGCAGTCCATAATCCATTAG | qRT-PCR |
| 3008-QR | AATTAGCCGCATAGCTGGCAACCG | qRT-PCR |
| 1726-QF | TCGGTGAAGTAGCGGCATTTGTCT | qRT-PCR |
| 1726-QR | ATCGCGCAGCCTAATAACCACGCA | qRT-PCR |
| 0506-QF | GCTCGCCCTGGTATGAAAGTCTTA | qRT-PCR |
| 0506-QR | TCGGCTAACACCACTTCACCTTTA | qRT-PCR |
| 0310-0311-RTF | CAATGCCATCAATGAGGATCTAAA | qRT-PCR |
| 0310-0311-RTR | CCAAATAAGATTGAGTGTGGCTGT | qRT-PCR |
| 0308-0309-RTF | CGGGCACTGAAGATAGTAAATTTT | qRT-PCR |
| 0308-0309-RTR | CAAGCTAAGCGAAGTTAAGATCAT | qRT-PCR |
| 16S QF | GCAGGCGGTTTGTTAAGCGAGATG | qRT-PCR |
| 16S QR | CTTCGCCACCGGTATTCCTCCAGA | qRT-PCR |

**Table S2 qRT-PCR system**

| **Component** | **Volume** |
| --- | --- |
| 2×Mix (SYBR) | 10 µL |
| Upstream primer | 1 µL |
| Downstream primer | 1 µL |
| ROX Reference Dye | 0.5 µL |
| cDNA template | 2 µL |
| ddH_2_O | 5.5 µL |

**Table S3 qRT-PCR reaction program**

| **Temperature** | **Time** | **Cycle number** |
| --- | --- | --- |
| 95℃ | 10 min | 1 |
| 95℃ | 10 s | 40 |
| 60℃ | 30 s |  |
| 95℃ | 1 min | 1 |
| 40℃ | 1 min | 1 |

**Table S4 Sequences of proteins encoded by the *men* gene cluster**

| **Protein name** | **Sequence** | **Sequence identity*** |
| --- | --- | --- |
| MenA | MNPWILAIRPRTLPAAIGPLLIGNILALHLNSFSWLIAATSMLCAILLQVAVNLANDYFDFKNGIDTAERIGPIRVTQSGMIPANSVRNAMILCLILALTVGSYLIWHGGWPIAILAAAAILGALGYSGGPYPLASHGLGEVAAFVFFGLVAVVGSYYLQAGDTTINAWLLGCAIGLFNAAIMLVNNTRDIRTDTQAGKHTLAVRIGEAQAKVLYQALIYLPFGLVIAGFLFGILPGLPVLLAGLSLLIARKLSSDFYAVSGEALNPLLGRTAKLTMIFSTLFSVGLVFTA | 96.56% |
| MenB | MTQQVSDIFDPTLWDEVSGFNFDDITYHRAKAHGTVRIAINRPECLNAFRPKTVDELYIALDHARQWSDVGCVLLTGNGPSAKGQYSFSSGGDQRIRGKDGYKYEGAEEGKADQARMGRLHILEVQRLIRFMPKVVIAVVPGWAVGGGHSLHVVCDLTLASKEHAIFKQTDPDVASFDSGYGSAYLAKMIGQKRAREIFFCGFNYSADEAFAMGMVNKSVPHAELETEALMWAKEINSKSPTAMRMLKYGFNMTDDGMVGQQLFAGEATRLAYASAEAQEGRDAFLEKRDQDFSAFPWHY | 96.33% |
| MenC | MILTSLSLYQYRLPLEVLLPVGKQRIDHRAGLVLQAFASANDRDTQVEVDIAPLSGIDIEQHDILGFSRENLAQVRSALTALLPQLCGQHIDKLIDFSEQTPYPSLAFGLSLLHAKLMGKLATVRPQNSTVPLIYHPTDASKALLANKIAHLGKDVRSVKVKVAQTSIEDELSLIYGILSTRPDIKLRLDANRGFSLEQAITFAACLPLEAIEYIEEPCHNPRDNPAFYQAIGLPFALDESLNDPEYQFEMYEGLAAIIIKPMLLGSLEKLSQLIDIAQNHGVRCILSSSLESSLGIRELAHLATILTPDETPGLDTLNAFSQDLIVSSGKTQCLTLSQLELIAQHTTHAKTNTDNELAEAKGE | 65.89% |
| MenD | MRTENTATLNLIWGALILEELARIGVQHVCMAPGSRSTPLTLAAAQQTKLKRHLHFDERGLGFMALGLAKASRAPVAIITTSGTAVANLYPAIVEAWLTHVPLIVLSGDRPPELLDCGANQAIVQPGIFAHYAKQINLPTPDAHIAPQALLTTIDEAVANQTRPVHINCMYREPLYPSELGVPILDTESPYLKPLQTWLQLSRPYTQYGKYKQVSNPSDDAIMRFVHGKGVIVAGTLTPEQDPQQLIALSQKIGWPLLTDAQSQLRQHPAAIGNIDQLLQHPKARNLLQEADRVLVFGGRLLSKRLISYLAEQHWHSYWQVLPEQNRLDPSHNAKHVWHANAAQFAALNWYRSSSANWANTLITYNDELHNLFVRNIDHGEFGEAQVVRAIANTRPLEQQLFIGNSLPVRLYDMYAPVSCCTATTYTNRGASGIDGLLATACGLAAHEGKPTSLIIGDLSQLHDLNSLAIAKSLASPLVIVILNNDGGNIFNLLPVPNEQVRNDYYRLSHGLEFGYAAAMFNLPYNQVDNLADFQDSYNEALDFQGASIIEVNVSQTQASDQIAELNLWVKQS | 91.27% |
| MenE | MLFPPTAISPLHQAARDFPQQTAITLAGKSINYQTLSQQVIALGEQLTALGIKEGNPVACISINNLEMICLYWACINIGAIFFPISPRFPTTQVQALIDTHQVGYYWSPTPDSALRHCSPLELQFNLLSTQSAKSIDFTRPSNVILTSGSSGFPKAAVHCLANHIANAEGARCVIPLEKGDAWLLSLPLFHIGGLAILNRCALVGASVAMPDPVISLNQQITRDKLTHVSLVPAQLIKLLSDENGKLESIKALLLGGGAISTDLLNQLKLRNIASFTSYGMTEMGSQITTGSALSDGSSGKLLPKRELKIADGVIWVRGECLFMGYLTADGIQKCVDKAGWFYTKDRGEWDANGNLHILGRVDNMFICGGENIQPEEIEAALKQHPLIDDAIVFAEPDAQFDHLPAAIVRGKITEDMTTIATELEPFLADKIARFKRPRHYYPWPEDSEQMGLKVNRKSIVINVKSNKKTGK | 67.08% |
| MenF | MPVHALSKDLKSLIDKLIQLKQVSPTEPIVQLSITTVSMPLISWLASQTQYPRIYWHGRDRIEEVAAIGACKDFRFETGVDDQQLAMVYQQQRALSTNQDIRYYGGVAFDRTIESWPEFGNSRFVLPRIEFRRSGTHFSLRVNLNFADNAPHTEIDNAIAAIEAVMPAKPLAPPTKLALLGRSDRPDFQRWKTLVEQVVDPKFNADTPKVVLSRLTQLEVNEPVDPWMVLACWQGRNPNSFQFGFQFSPERTFISCSPERLFRRRQQELFTEALAGTTVRGLNQEEDIALANALLEDNKNSVENQLVRSHIVNMLTPLSHYVGADELATIFKLNHIQHLHRAIRAELKAGVNDFQLLQALHPTPAVGGLPRHSAMNFIRQREGYMRGWYAGACGYFNKYESEFSVAIRCTLIEPGRINLFAGAGIIAGSDPEAEWQELENKLATIMSILIEL | 86.95% |
| MenG | MSEGESKSTHFGYKTVEADKKAELVAGVFHSVAAKYDIMNDVMSFGIHRFWKRHTIEVSGARPGMKVLDLAGGTGDLTAKFSHLVGDKGEVVLADINDSMLKVGRTKLRDKGIVNNVSYVQANAEALPFPDNHFDIITIAFGLRNVTDKDAALRSMNRVLKPGGKLLVLEFSKPQHEIMRKVYDLYSFKVLPKMGELITKDADSYEYLAESIRMHPDQDTLKQMMVDAGFEQVDYTNMTDGIVALHRGYKF | 97.61% |
| MenH | MPTMARYGETSLPVLVLLHGFLGTKADWLPLMPVLSQHFHCICVDLPGHGDNQDELAPTELEGFSQCVENVIAHLDGLHIGQFHLYGYSLGGRIALHLANTYPQRLLSLTLESCHPGLDDLEEKTTRASNDALWADRLMRLSSKEFLALWYQQAVFADMGTTKRQSLIDLRTKALDQHPKQKLQQIYLATSLAKQASLWNVPSQLTCECHFFAGTEDSKFSAIAEKWQQQAPILLHSIHGAGHNIHQMAPAALISKIVALLG | 62.41% |
| PaaI | MRTENTATLNLIWGALILEELARIGVQHVCMAPGSRSTPLTLAAAQQTKLKRHLHFDERGLGFMALGLAKASRAPVAIITTSGTAVANLYPAIVEAWLTHVPLIVLSGDRPPELLDCGANQAIVQPGIFAHYAKQINLPTPDAHIAPQALLTTIDEAVANQTRPVHINCMYREPLYPSELGVPILDTESPYLKPLQTWLQLSRPYTQYGKYKQVSNPSDDAIMRFVHGKGVIVAGTLTPEQDPQQLIALSQKIGWPLLTDAQSQLRQHPAAIGNIDQLLQHPKARNLLQEADRVLVFGGRLLSKRLISYLAEQHWHSYWQVLPEQNRLDPSHNAKHVWHANAAQFAALNWYRSSSANWANTLITYNDELHNLFVRNIDHGEFGEAQVVRAIANTRPLEQQLFIGNSLPVRLYDMYAPVSCCTATTYTNRGASGIDGLLATACGLAAHEGKPTSLIIGDLSQLHDLNSLAIAKSLASPLVIVILNNDGGNIFNLLPVPNEQVRNDYYRLSHGLEFGYAAAMFNLPYNQVDNLADFQDSYNEALDFQGASIIEVNVSQTQASDQIAELNLWVKQS | 95.86% |
| 0311 | MHISNMDGLDILSLKILVSLYENKSATYVSKALDIPAPKISRSLKNLREIFGDELFIRRKYGLFPNEFATHLYPIAKEAIECTDKFQKARMANTRQVKTHVEIAAPGLIAYAFPKALMQAIKEEQKPMHININPWSMHTTQDIINGDITLGICCNKSAADVAAFGDKLLTTELHRMDKLYLIASRHHPIFKREITLETIAEYPYVNTDLGNPSTKLSPYQEFCITNGIKLNTEITITSISSLFEYLGESNAVSLLPYRIVYEMVSDIPELHTCQLSQYEAERLYADTEAPTIFLVQKNQPNAINEDLKWLSGEIQKIVDRII | 23.74% |

***** The data are based on sequence alignments of these proteins with their *S. oneidensis* MR-1 homologues.
