# Supplementary material for: Menaquinone synthesis genes are critical for the decolorization of acid yellow 36 in Shewanella putrefaciens CN32
Source: Microbiol Spectr. 2026 Sep 2;14(10):e01996-26. doi: 10.1128/spectrum.01996-26 (PMC13688807; doi:10.1128/spectrum.01996-26)
Supplement: Supplementary file 3 [file spectrum.01996-26-s0008.docx]

**Figure S1** Decolorization capacity of mini‑Tn5 insertion mutants.

**Figure S2** Sequence alignment of Sputcn32_0310 from *S. putrefaciens* CN32 with MenD from *S. oneidensis* MR-1.

**Figure S3** Sequence alignment of Sputcn32_1269 from *S. putrefaciens* CN32 with LutB from *S. oneidensis* MR-1.

**Figure S4** Effect of *menD* overexpression on decolorization.

**Figure S5** Co‑transcription analysis of *Sputcn32_0311* with *menD*, *menH*, *menC*, and *menE*. (A) Schematic of the operon structure containing *Sputcn32_0311*. (B) Amplification of intergenic regions from cDNA of this gene cluster.

**Figure S6** Effects of sodium chloride and glucose on biofilm formation of *S. putrefaciens* CN32.
